# Predictors of Food Insecurity among Australian University Students: A Cross-Sectional Study

**DOI:** 10.3390/ijerph17010060

**Published:** 2019-12-19

**Authors:** Megan C. Whatnall, Melinda J. Hutchesson, Amanda J. Patterson

**Affiliations:** 1 School of Health Sciences, Faculty of Health and Medicine, University of Newcastle, Callaghan 2308, NSW, Australiamelinda.hutchesson@newcastle.edu.au (M.J.H.); 2 Priority Research Centre for Physical Activity and Nutrition, University of Newcastle, Callaghan 2308, NSW, Australia

**Keywords:** food insecurity, food security, university students, college students

## Abstract

Food insecurity is much higher among university students than the general population, and is linked with poorer mental health, diet and academic achievement. The aim of this study was to explore the level of food insecurity among a sample of Australian university students and determine which socio-demographic and student characteristics predict food insecurity. An online cross-sectional survey with students from the University of Newcastle, Australia was conducted in 2017–2018. Food insecurity was assessed using the 6-item US Department of Agriculture Food Security Survey Module, and socio-demographic (e.g., age, living situation) and student characteristics (e.g., undergraduate/postgraduate student) were captured. Multivariate logistic regression was used to determine the odds of food insecurity for each of the socio-demographic and student characteristics, and included characteristics of significance in bivariate analyses as potential confounders. Data for 366 students were analysed (mean age 27.3 ± 10.4 years, 27.3% male). Forty-eight percent of participants were food insecure. The odds of food insecurity were higher among students living in rental accommodation compared with their parents’ home (OR = 2.39, 95% CI 1.41, 4.06), and undergraduate compared with postgraduate students (OR = 3.50, 95% CI 1.83, 6.69). Commencing university and moving away from parents may be key times for intervention. Strategies that can provide longstanding benefit are needed to address the high level of food insecurity among university students.

## 1. Introduction

Food security is where “all people at all times have physical and economic access to sufficient, safe and nutritious food to meet their dietary needs and food preferences for an active and healthy life“ [1]. Food insecurity, i.e., the absence of this, is a widespread issue among university students internationally [2]. A recent systematic review of 18 studies among university students from five countries found an average rate of food insecurity of 42% [2], with the majority of these studies in western countries, including nine in the USA and three in Australia. The reported rates of food insecurity among university students is much higher than the general population. For example, 12% of USA households were reported to be food insecure in 2017 [3], 10% of adults in the UK were reported to be food insecure in 2014 [4] and 5% of Australians were reported as food insecure in 2012 [5]. The impact of food insecurity among university students is significant. It has been associated with poorer mental health, including higher odds of having symptoms of depression and lower self-rated mental health status [6,7], poorer diet, predominantly lower fruit and vegetable intakes, as well as lower odds of consuming breakfast and home cooked meals [8,9], and with poorer academic achievement, including lower GPA scores and higher odds of failing or withdrawing from courses [6,10,11].

In terms of accurately assessing food insecurity, this is highly dependent on the tool used in assessment [12,13]. In Australia the single item measure historically used to assess food insecurity has been found to underestimate prevalence when compared with longer and more comprehensive measures such as the United States Department of Agriculture Food Security Survey Modules (USDA FSSM) [12]. For example, Hughes et al. assessed food insecurity among a sample of 399 Australian university students using both the standard Australian single item measure and a multi-item measure adapted from the USDA FSSM, and reported rates of 12.7% and 46.5% respectively [13]. The variability in these findings alludes to the multifaceted nature of food insecurity, highlighting the importance of using comprehensive and sensitive tools to assess food insecurity, as well as the importance of exploring characteristics which predict food insecurity.

Studies exploring prevalence and predictors of food insecurity are key, as they provide information for the development of strategies to address the issue, as well as a system of monitoring and surveillance [13,14]. However, the majority of studies to date have been conducted in USA student samples [2], with few studies among Australian university students. A 2019 systematic review of food insecurity in Australia identified four of 57 studies were in university students, with the most recent conducted in 2014 [15]. Given the differences between countries in various factors associated with food insecurity, some international findings are not necessarily generalisable to the Australian setting. For example, where students live has been shown to be associated with food insecurity status and the typical living situation of students differs between countries, with USA students typically living in university accommodation where Australian students more often live off-campus in the parental home or in shared rental accommodation [16,17]. The aim of the current study was to explore the level of food insecurity among a sample of Australian university students using a comprehensive measure (USDA FSSM), and determine which socio-demographic and student characteristics predict food insecurity.

## 2. Materials and Methods

### 2.1. Study Design

This study was a cross-sectional analysis of data from an online survey conducted at the University of Newcastle (UON) in 2017–2018 using Qualtrics (https://www.qualtrics.com/). The primary aim of the survey was to determine students’ dietary intake and opinions about the cost and availability of foods and beverages at UON campuses. It also included a measure of food security status. The conduct and reporting of this study is compliant with STROBE guidelines [18].

### 2.2. Participants and Recruitment

Participants were students from two campuses at the UON, including the Newcastle main campus and the Ourimbah campus, both located in regional areas in New South Wales (NSW), Australia. All students enrolled in face-to-face courses at either campus for university semester 2 2017 were eligible to participate (~28,000). Recruitment was staged, firstly an email invitation was sent to students who had completed a previous online survey in 2017 (*n* 2803) and indicated their interest in being re-contacted for future research (*n* 1582), including a reminder email one week later. Secondly, students were made aware of the study via the University’s social media pages, and posters and computer screensavers displayed on campus, with these used for one month within the survey dates, and members of the University Health Promotion Working Group were contacted twice via email, in November and March, with the request to disseminate study details to students. The University Health Promotion Working Group is a consultative group of university staff and students who guide health promotion initiatives at UON, including health professionals, health academics, staff from key divisions (e.g., Student Residences, Student Communications and Marketing) and student organisation executive. The survey was open from the 17th October 2017 to 12th March 2018. On survey completion participants could choose to enter a prize draw, including ten gift vouchers valued at $AU 100. All participants gave informed consent prior to completing the survey.

### 2.3. Measures

#### 2.3.1. Socio-Demographic and Student Characteristics (Independent Variables)

Socio-demographic data collected included age, gender, country of birth, usual language spoken at home, Aboriginal or Torres Strait Islander (ATSI) origin, marital status, living situation, who they live with, sources of financial support (parents/guardians, partner, government, scholarship, other or none) and hours of paid work. Student characteristic data collected included type of degree (undergraduate, postgraduate (research or coursework), enabling course (i.e., transition to university courses for students not meeting direct entry admission criterion) or English language course for international students), faculty of study, number of years studying and whether they were a domestic or international student.

#### 2.3.2. Food Insecurity (Dependent Variable)

Food security status was assessed using the USDA FSSM 6-item short form [19], which focuses on economic access to food. The six items include, *“The food that I bought just didn’t last and I didn’t have enough money to get more”, “I couldn’t afford to eat balanced meals”, “In the last 12 months did you ever cut the size of your meals or skip meals because there wasn’t enough money for food?”* and if so, *“How often did this happen?”, “In the last 12 months did you ever eat less than you felt you should because there wasn’t enough money for food?”,* and *“ In the last 12 months were you ever hungry but didn’t eat because there wasn’t enough money for food?”.* Affirmative responses were attributed a score of one, with the total food security score calculated as the sum of individual responses, ranging from 0–6. Food security status was then categorised as; high/marginal food security (0–1), low food security (2–4) and very low food security (5–6). The 6-item USDA FSSM was chosen as the measure of food security as it is a more sensitive measure in terms of estimating prevalence and severity of food insecurity than the Australian single item measure [12], and because a validated multi-item measure for the Australian population is not yet available [20].

### 2.4. Statistical Analysis

Statistical analysis was conducted using Stata statistical software version 14.1. Socio-demographic and student characteristics and food security status are described as percentages for categorical variables and means and standard deviations (SD) for continuous variables. A total of 513 individuals consented to participate in the survey, of which 437 were eligible. Of these, 366 were included in this analysis, as 71 were excluded due to missing data on food security status. For the purpose of further analyses, food security status was dichotomised as food secure (high/marginal food security) or food insecure (low food security and very low food security) in accordance with the USDA FSSM 6-item short form guide [19]. Bivariate associations between food security status (dependent variable) and socio-demographic and student characteristics (independent variables) were explored using Chi-square tests/Fisher’s exact test for categorical variables, depending on frequency per category, and t-tests/Wilcoxon Mann–Whitney tests for continuous variables, depending on the normality of the data. A multivariate logistic regression model was used to determine the odds of being food insecure for each of the socio-demographic and student characteristics, including the characteristics of significance in the bivariate analyses to account for potential confounding. For the multivariate model the variable living situation was modified to combine the categories boarding/homestay and irregular due to low frequency within these categories. Multivariate logistic regression results are reported as odds ratio (OR) and 95% CI. A multivariate logistic regression model was used to suit the data (i.e., dichotomous dependent variable) and as this approach has been used in similar previous studies [17]. Statistical significance was set at *p* < 0.1 for the identification of potential confounders (i.e., the bivariate analyses), and *p* < 0.05 for the logistic regression.

## 3. Results

### 3.1. Participant Characteristics

Participants mean age was 27.3 years and just over a quarter (27.3%) were male (Table 1). The majority were born in Australia (85.5%), while 2.5% were of Aboriginal or Torres Strait Islander origin. The largest proportion were living in rental accommodation (43.7%) or their parents’ home (35.3%). Participants were from all five faculties across the university and represented students across the range of number of years spent studying, with 37.2% in their first year of study and 23.2% in their fourth year or later (Table 2).

### 3.2. Food Insecurity

Overall, close to half of the participants (48.1%) were categorised as food insecure, including 24.9% having low food security and 23.2% having very low food security. For the individual items in the USDA FSSM, 45.4% of participants reported *“often true”* or *“sometimes true”,* to the statement *“The food that I bought just didn’t last and I didn’t have enough money to get more”,* while 48.1% reported *“often true”* or *“sometimes true”,* to *“I couldn’t afford to eat balanced meals”.* Over a third of participants (43.7%) reported that within the last 12 months they had cut the size of their meals or skipped meals because there was not enough money for food, and of these, 29.9% reported that this occurred *“almost every month”.* Almost one third of participants reported yes to the statements *“In the last 12 months did you ever eat less than you felt you should because there wasn’t enough money for food?”,* (32.8%) and *“In the last 12 months were you ever hungry but didn’t eat because there wasn’t enough money for food?”* (27.1%).

### 3.3. Associations between Socio-Demographic and Student Characteristics and Food Security Status

In the bivariate analyses, food security status was significantly associated with Aboriginal or Torres Strait Islander origin (*p* = 0.071), marital status (*p* = 0.039), living situation (*p* < 0.001) and type of degree (*p* = 0.001) (Table 1 and Table 2). The proportion of students who were food insecure was significantly higher among students who were of Aboriginal or Torres Strait Islander origin (77.8%) compared with non-ATSI students (47.3%). In terms of marital status, the highest rate was among students who were separated/divorced/widowed (64.7%) with the lowest rate among students who were married (31.5%). Students who were living in irregular (80.0%) and boarding/homestay (75.0%) accommodation had the highest rates of food insecurity while the lowest was among students living in their own home (23.3%), and undergraduate students (52.9%), and students enrolled in enabling courses (50.0%) had higher rates compared with postgraduate students (26.6%).

In the multivariate logistic regression model, controlling for all socio-demographic and student characteristics found to be significant in bivariate analyses, living situation and type of degree remained significantly associated with food security status (Table 3). In comparison with students living in their parents’ home, students living in rental accommodation were 2.4 times more likely to be food insecure (OR = 2.39, 95% CI 1.41, 4.06, *p* = 0.001). Students living in on-campus residences (OR = 2.27, 95% CI 0.92, 5.60, *p* = 0.074) and boarding/homestay or irregular accommodation (OR = 4.59, 95% CI 0.91, 23.29, *p* = 0.066) were also more likely to be food insecure compared with students living in their parents’ home, however these findings did not reach significance. Compared with postgraduate students, undergraduate students were 3.5 times more likely to be food insecure (OR = 3.50, 95% CI 1.83, 6.69), while students enrolled in enabling courses were three times more likely to be food insecure (OR = 2.99, 95% CI 1.12, 7.96).

## 4. Discussion

This cross-sectional study identified high rates of food insecurity among a sample of Australian university students. Close to half of the sample were identified as food insecure, which is comparable with other university student samples in Australia and overseas [2], while substantially higher than the general Australian population rate of approximately 5% [5]. Students living in rental accommodation were at higher odds of being food insecure than students living in their parents’ home, and undergraduate students were at higher odds than postgraduate students. The study findings provide important insight to inform future strategies to address food insecurity among university students, in terms of identifying at-risk students and key time points for intervention.

In the current study, 48.1% of university students were food insecure. This rate of food insecurity is slightly higher than the average rate of 42% reported in the systematic review of food insecurity prevalence in university students internationally [2], and at the higher end of the range of prevalence from studies included in the review conducted within Australian universities (26–48%) [13,17,21]. A noteworthy point here is that these previous studies conducted in Australian universities are from 2011 to 2014, therefore suggesting that the rate of food insecurity has been consistently high for a number of years. The most recent national data on the Australian population found that a comparatively lower 5% had experienced food insecurity in the previous 12 months [5]. However, the single item measure used to collect this data has been shown to underestimate the true rate of food insecurity [5,12]. The main items contributing to participants’ food insecurity in the current study were not being able to afford to eat balanced meals, food purchases did not last and there was not enough money to purchase more, and the need to cut the size of meals or skip meals because there was not enough money for food. In a US study by Payne-Sturges et al., the main contributors were similar, including inability to eat balanced meals, eating less and being hungry due to lack of money for food [7]. However, other studies in Australian university students using one of the USDA FSSM have not reported findings for the individual items, therefore further comparisons cannot be made. The findings of the current study provide evidence that Australian university students, similar to those in other western countries, are an at-risk group for food insecurity. This difference between university students and the general population could be driven by the fact that university students compared with their non-studying peers have additional study-related expenses, and lower income due to less time to work and having lower paid jobs due to lower skills.

The characteristics that were significantly associated with food insecurity status in the current study were living situation and whether students were studying undergraduate or postgraduate degrees. Students who lived in rental accommodation were 2.4 times as likely to be food insecure than students who lived in their parents’ home. This is similar to the findings of Gallegos et al. among a comparable Australian sample, where students who were renting were 3.4 times as likely to be food insecure than those living in their parents’ home [17]. Studies from the USA also report similar findings, for example higher rates of food insecurity were found among USA community college students who were living alone, with a partner or with roommates compared with those living with parents or relatives [10]. While there are some mixed findings around living situation and food insecurity among this group, for example between students who live on campus versus off-campus in USA students [22,23], the consistent finding seems to be that students who live with their parents are less likely to be food insecure than most other living situations. For students who live within the parental home, living expenses are likely lower and/or food more readily available, therefore contributing to the lower likelihood of food insecurity. Therefore, universities aiming to address food insecurity should target programs or strategies to those students not living with their parents, while preventative strategies should be targeted to students during their transition to living away from parents for the first time (e.g., at commencement of university study).

It was also found in the current study that students enrolled in undergraduate degrees and enabling courses, i.e., transition to university courses for students not meeting direct entry admission criterion, had higher odds of being food insecure than postgraduate students. This is consistent with a study of 692 USA students by Hagedorn et al., where freshman were almost three times more likely to be food insecure than graduate students [23]. The higher food insecurity among undergraduates and enabling course students could be related to lower educational attainment as a factor of income, in that postgraduate students have a higher level of educational attainment, which would enable them to work in more skilled employment and therefore earn a higher rate of pay. Additionally, for students enrolled in enabling courses the higher rate of food insecurity could be in part explained by lower socio-economic status, a known predictor of food insecurity [21,22]. Enabling course students are more likely to come from a lower socio-economic status background than other students as disadvantage, such as socio-economic disadvantage, is one of the possible admission criteria. In support of this are the findings of a USA study of 354 college students from Patton-Lopez et al., where an income of <$US 15000 compared with ≥$US 15000 was associated with a two times higher odds of being food insecure [22]. The indicators of financial status used in the current study, the number of hours students worked and whether they received financial support, were not significantly associated with food insecurity, however, measuring income directly may have shown a different result. Overall, these findings suggest that when students commence university for the first time may also be a key time to implement preventative strategies for food insecurity, and that strategies should particularly target students from lower socio-economic backgrounds.

In terms of implications, the evidence of such high food insecurity should be used in advocating for universities to address the issue within the broader context of University health promotion. Currently the most widely used strategy to support students experiencing food insecurity is food pantries or food vouchers [24], solutions which provide short-term relief, and there is a need for strategies that can provide longstanding benefit and which recognise that to be food secure is a fundamental human right [17]. For example, strategies such as interventions to improve students’ food literacy and ability to manage their finances stand to provide lifelong knowledge and skills, and therefore long-term benefit [2,17]. At the broader level, there is a need for strategies which overall work towards creating a health promoting university environment [25], including strategies which target individuals, e.g., financial advisory services, and health promotion initiatives to improve food literacy, and strategies which target the environment, e.g., ensuring access to food on-campus that is healthy and affordable. Further, there is a need for strategies which ensure adequate support to students for factors such as housing and healthcare, which potentially extend beyond the responsibility of universities to others such as Government. In terms of future research in this area, studies should explore how changes in food security status impact health and academic outcomes over time, for example cohort studies and intervention studies.

The strengths of this study include the use of the 6-item USDA FSSM as a measure of food security status, as a more comprehensive and sensitive measure than the Australian single item measure [12,19], as well as considering a broad set of student and socio-demographic characteristics as potential predictors of food insecurity. In terms of limitations, not all potential predictors of food insecurity were assessed, for example food availability and income. In addition, the 6-item USDA FSSM focuses on economic access to food, which is only one component of food security. Other contributors to food security, including physical accessibility of food, are not considered by the tool, but are potentially relevant to the study population. Further, the data collection period which allowed completion during and outside university semesters may have influenced responses to some socio-demographic characteristics (e.g., hours of paid work, financial support and living situation). Other limitations to consider include the cross-sectional study design, the fact that the sample was a small proportion of the eligible UON student body (1%), and that recruitment was via convenience sampling and therefore not a random sample, which may limit the generalisability of findings and representativeness of the sample. In terms of representativeness, the proportion of female, undergraduate and ATSI students was slightly higher than the average across Australian universities [26]. Despite the lower proportion of males in the sample, this is consistent with other research of university students [21], and reflects the fact that a lower proportion of males than females attend university, and of the established challenges in recruiting males to health research studies including that they are less likely than females to volunteer [27].

## 5. Conclusions

In summary, food insecurity is highly prevalent in university student populations, and it is in the interest of individuals and universities to address this given the impacts of food insecurity on health and academic outcomes. At the individual level, intervention strategies are needed which can provide long-term benefit such as strategies to improve students’ food literacy and skills in managing their finances, as a supplement to current strategies offering short-term relief such as food pantries. Moreover, environmental level strategies should be considered which address factors such as income and housing support. Key times for intervening may be when students commence university and when they first move away from parents, as this study found higher rates of food insecurity among undergraduate students and students not living with their parents. Intervention strategies and support should also be ongoing, as individuals should at all times be food secure. Additionally, future research is needed to explore the changes in, and impact of, food insecurity over time among university students.

## Figures and Tables

**Table 1:** Socio-demographic characteristics of a sample of Australian university students by food security status (*n* = 366).

Variable	Food Secure (*n* = 190)	Food Insecure (*n* = 176)	Total (*n* = 366)
**Age (years) (mean ± SD)**	28.5 ± 12.1	26.0 ± 8.0	27.3 ± 10.4
**Gender % (*n*)**			
Female	71.6 (136)	70.5 (124)	71.0 (260)
Male	26.8 (51)	27.8 (49)	27.3 (100)
Another gender identity	1.6 (3)	1.7 (3)	1.6 (6)
**Country of birth % (*n*)**			
Australia	83.2 (158)	88.1 (155)	85.5 (313)
Other	16.8 (32)	11.9 (21)	14.5 (53)
Aboriginal or Torres Strait Islander % (*n*) *			
Yes	1.1 (2)	4.0 (7)	2.5 (9)
No	99.0 (188)	96.0 (169)	97.5 (357)
**Language spoken at home % (*n*)**			
English	91.1 (173)	91.5 (161)	91.3 (334)
Other	9.0 (17)	8.5 (15)	8.7 (32)
Marital status % (*n*) *			
Never married	69.0 (131)	75.0 (132)	71.9 (263)
Married	19.5 (37)	9.7 (17)	14.8 (54)
De facto	8.4 (16)	9.1 (16)	8.7 (32)
Separated/divorced/widowed	3.2 (6)	6.3 (11)	4.6 (17)
Living situation % (*n*) *			
Own home	17.4 (33)	5.7 (10)	11.8 (43)
Parents home	40.0 (76)	30.1 (53)	35.3 (129)
On-campus residences	5.3 (10)	8.5 (15)	6.8 (25)
Renting	36.3 (69)	51.7 (91)	43.7 (160)
Boarding/homestay	0.5 (1)	1.7 (3)	1.1 (4)
Irregular	0.5 (1)	2.3 (4)	1.4 (5)
**Living with % (*n*)**			
Parents	21.1 (40)	13.1 (23)	17.2 (63)
Parents & others	18.4 (35)	17.6 (31)	18.0 (66)
Partner	15.3 (29)	13.1 (23)	14.2 (52)
Partner & children	11.6 (22)	8.5 (15)	10.1 (37)
Children	3.7 (7)	6.3 (11)	4.9 (18)
Other adults	16.8 (32)	24.4 (43)	20.5 (75)
Other combination not including Parents	5.3 (10)	5.1 (9)	5.2 (19)
None of the above	7.9 (15)	11.9 (21)	9.8 (36)
**Paid work (hours/week) (mean ± SD)**	10.4 ± 11.0	10.5 ± 11.4	10.5 ± 11.2
**Receiving financial support % (*n*)**			
Yes	74.7 (142)	69.3 (122)	72.1 (264)
No	25.3 (48)	30.7 (54)	27.9 (102)

* Indicates statistically significant difference in food insecurity status at *p* < 0.1 level.

**Table 2:** Academic characteristics of a sample of Australian university students by food security status (*n* = 366).

Variable	Food Secure (*n* = 190)	Food Insecure (*n* = 176)	Total (*n* = 366)
Type of degree % (*n*) *			
Undergraduate	67.4 (128)	81.8 (144)	74.3 (272)
Postgraduate	24.7 (47)	9.7 (17)	17.5 (64)
Enabling course ^a^	7.9 (15)	8.5 (15)	8.2 (30)
**Domestic/International % (*n*)**			
Domestic	91.6 (174)	94.9 (167)	93.2 (341)
International	8.4 (16)	5.1 (9)	6.8 (25)
**Number of years studying % (*n*)**			
1st year	36.8 (70)	37.5 (66)	37.2 (136)
2nd year	14.7 (28)	16.5 (29)	15.6 (57)
3rd year	23.7 (45)	24.4 (43)	24.0 (88)
4th year	14.2 (27)	114.8 (26)	14.5 (53)
5th year or later	10.5 (20)	6.8 (12)	8.7 (32)
**Faculty of study % (*n*)**			
Business and Law	4.2 (8)	3.4 (6)	3.8 (14)
Education and Arts	26.8 (51)	33.0 (58)	29.8 (109)
Engineering and Built Environment	20.0 (28)	13.1 (23)	16.7 (61)
Health and Medicine	29.5 (56)	25.6 (45)	27.6 (101)
Science	12.6 (24)	18.2 (32)	15.3 (56)
English Language and Foundation Studies Centre	6.8 (13)	6.8 (12)	6.8 (25)

^a^ Enabling courses are transition to university courses for students not meeting direct entry admission criterion. * Indicates statistically significant difference in food insecurity status at *p* < 0.1 level.

**Table 3:** Multivariate logistic regression results of food security status with socio-demographic and student characteristics in a sample of Australian university students (*n* = 366).

Variable	Odds Ratio	95% CI	*p*-Value
**Aboriginal or Torres Strait Islander**	3.01	0.58, 15.56	0.188
**Marital status**			0.525
Reference category = never married			
Married	0.69	0.31, 1.51	0.345
De facto	1.01	0.44, 2.32	0.984
Separated/divorced/widowed	1.78	0.57, 5.57	0.319
**Living situation**			**0.005**
Reference category = parents’ home			
Own home	0.72	0.28, 1.90	0.514
On-campus residences	2.27	0.92, 5.60	0.074
Renting	2.39	1.41, 4.06	**0.001**
Boarding/homestay/irregular	4.59	0.91, 23.29	0.066
**Type of degree**			**0.001**
Reference category = postgraduate			
Undergraduate	3.50	1.83, 6.69	**<0.001**
Enabling course ^a^	2.99	1.12, 7.96	**0.028**

^a^ Enabling courses are transition to university courses for students not meeting direct entry admission criterion. Significant p-values are in bold.
